# Epithelial-Myoepithelial Carcinoma of the Parotid Gland: A Report of a Rare Case and Review of Literature

**DOI:** 10.7759/cureus.59701

**Published:** 2024-05-05

**Authors:** Prachi Surolia, Rajanikanth Kambala, Nitin Bhola

**Affiliations:** 1 Oral and Maxillofacial Surgery, Sharad Pawar Dental College and Hospital, Datta Meghe Institute of Higher Education and Research, Wardha, IND

**Keywords:** recurrence local, neoplasm recurrence, head and neck neoplasms, salivary gland carcinoma, epithelial-myoepithelial carcinoma

## Abstract

Epithelial-myoepithelial carcinoma (EMC) is a rare tumor, characterized by two different cell populations and both demonstrate a malignant nature microscopically. It constitutes less than 2% of all salivary gland malignancies. The World Health Organization (WHO) has classified this disease as a separate pathological category. The diagnosis of this tumor is arrived by biopsy. It shows slow growth and is small in size; it appears in ulcerative form of mucosa in some cases. Gland cells consist of two layers of outer myoepithelium cells and inner epithelial cells. Vimentin staining is positive. It shows calponin, muscle-specific actin, S100, smooth muscle actin, p63, and smooth muscle myosin heavy chain I. Examining different sets of data reveals that tumors exhibiting a solid growth pattern, nuclear atypia, DNA aneuploidy, and increased proliferative activity typically display a more aggressive nature, accompanied by a heightened likelihood of local recurrences and metastases. The clinical and radiological observations frequently resemble those of a benign tumor. Due to the uncommon nature of EMC, there is currently no established standard treatment protocol. It is considered a low-grade tumor where good resection holds better results. Individuals displaying histopathological indicators of aggressive disease should be evaluated for potential adjuvant radiotherapy. We present a case of a patient who had recurrence twice in a period of seven years despite surgical management, chemotherapy, and radiotherapy.

## Introduction

As early as 1956, epithelial-myoepithelial carcinoma (EMC) was identified under various names like adenomyoepithelioma, clear cell adenoma, tubular solid adenoma, and clear cell carcinoma, reflecting its wide range of histopathological features. The initial descriptions of biomorphic clear cell tumors, which displayed histological traits like EMC, were recorded in German studies [1]. Corridan identified a tumor rich in glycogen and classified it as clear cell adenoma [2]. This tumor consisted of enlarged cells with clear cytoplasm and dark nuclei, as well as cells with eosinophilic cytoplasm. Periodically, small ducts with a single layer of cuboidal epithelial cells showing eosinophilic cytoplasm were observed among large areas of clear cells. Contrary to Corridan's initial belief that it was a clear cell variant of pleomorphic adenoma, the photomicrographs and microscopic details suggest that the tumor was more accurately characterized as an epithelial-myoepithelial cell carcinoma [2].

Donath et al. in 1972 was the first to elaborate on EMC and was recognized first in the WHO classification, 1991 [3]. EMC constitutes nearly 1% of all salivary gland tumors [3]. Mutations of HRAS, AKT1, CTNNB1, and PIK3CA are seen. EMC has a five-year survival rate of 94%, and recurrence is local in 30%-50% patients [4]. According to various recent reports, data indicates that the primary site of origin for EMCs is commonly the parotid gland. Majority of the EMCs involve the parotid gland, secondly submandibular gland, and smaller salivary glands. There are cases reported in the breast, trachea, larynx, hypopharynx, and maxillary sinus [5]. The mean age of diagnosis for EMC is 60 years, with a female predominance; however, kids as young as eight years old have been diagnosed with EMC [6].

Although local recurrences are common, instances of distant metastases are rare, with only a limited number of documented cases involving the kidney and the lung. Many individuals with metastasis to the lung and the kidney ultimately succumbed to their illness, with the duration from diagnosis to death spanning from 18 months to 28 years [1]. Patients have succumbed to their condition following the occurrence of metastases in the kidney, brain, and lung. The metastasis in the kidney manifested post six local recurrences and 28 years post-parotidectomy where the metastases in the brain and lung were identified one to two years after the initial diagnosis [7,8,9]. Another report described a clear cell tumor that originated in the parotid gland of a four-year-old girl. The tumor recurred four times before her death at the age of 43. Metastases to the lung and retroperitoneal lymph nodes were apparent at the time of her demise [10].

Altogether, the information suggests that epithelial-myoepithelial cell carcinomas display a mild malignant potential, setting them apart from the more aggressive salivary gland duct adenocarcinomas that do not exhibit myoepithelial differentiation.

## Case presentation

A 56-year-old female reported to our center in February 2023 for swelling over infraorbital and upper back region of the jaw of the left side for 1.5 years approximately. She stated that the mass had been slowly increasing in size to the present size of approximately 5 x 4 cm for 1.5 years. She presented with a history of postoperative reduced mouth opening for 1.5 years approximately, a history of postoperative reduced salivation for 8-10 months approximately, a history of postoperative change in the consistency of saliva from thin to thick ropy for 6-7 months approximately, and a history of postoperative weight loss (4-5 kg) in the last one month. The case was diagnosed with "local recurrence of myo-epithelial carcinoma of minor salivary gland" in 2016 for which palate excursion was done and a partial maxillectomy in May 2016, after which the patient underwent 30 cycles of radiotherapy last August 2016 and was again operated in 2021 due to recurrence after which the patient received six cycles of chemotherapy from August to October 2021 and 10 cycles of radiotherapy last on October 2021. The patient had no other habit history than betel nut chewing 4-5 times a day for 15 years approximately.

**Figure 1:**
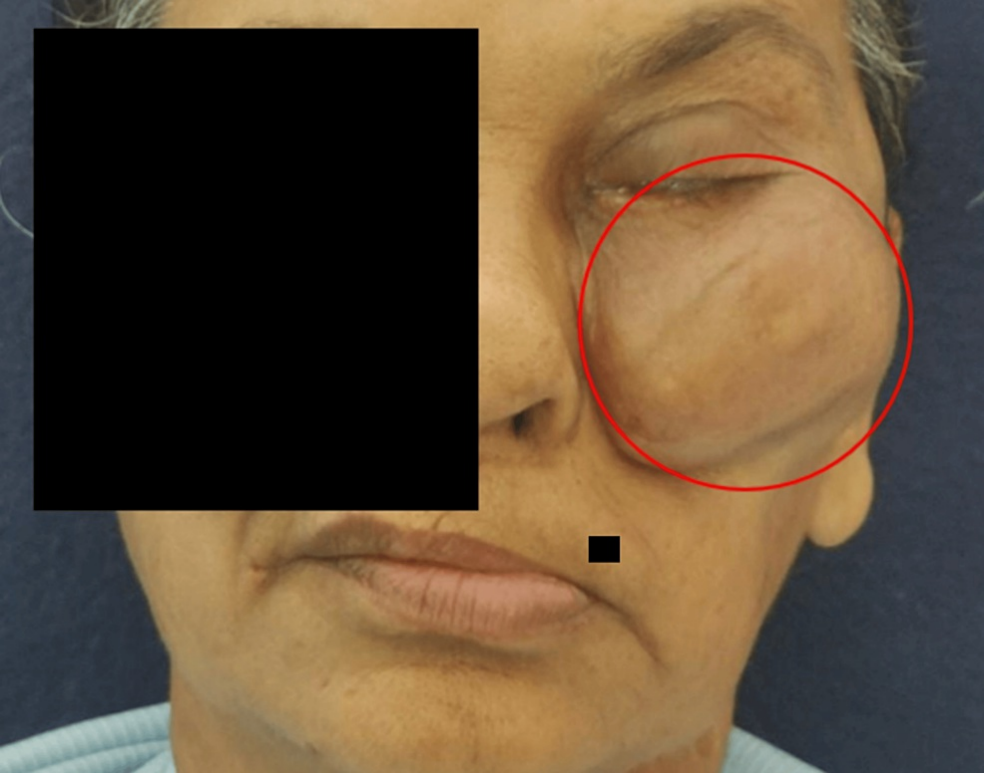
Extraoral clinical picture showing extension of the lesion supero-inferiorly from the infraorbital rim to the ala tragus line on the left side

On extraoral examination, the face showed gross asymmetry due to the presence of swelling over the left infraorbital and upper jaw region of size 6 x 4 cm approximately extending supero-inferiorly from infraorbital rim to ala tragus line on the left side (Figure 1).

**Figure 2:**
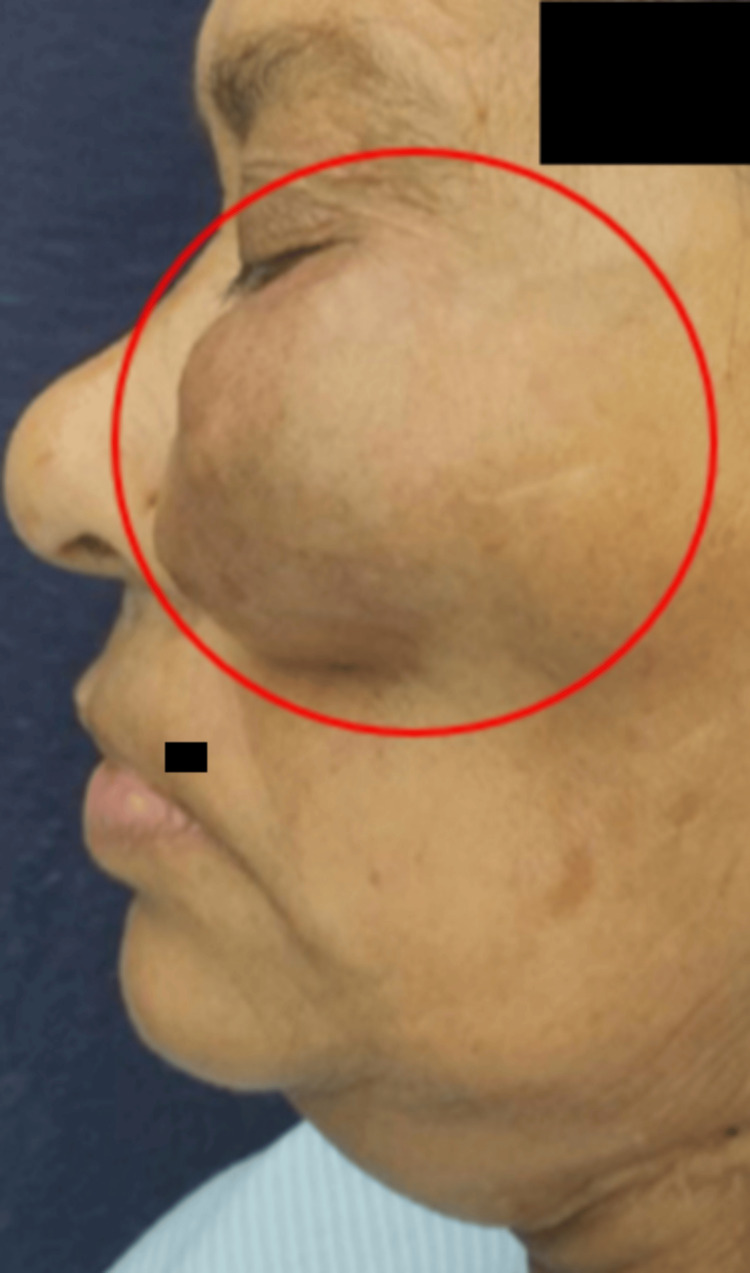
Left lateral view showing an extension anteroposteriorly extending from the ala of the nose to 3 cm short of the tragus of the left ear

The shape was roughly oval, the color was the same as the adjacent skin, borders were ill-defined, and the consistency was firm and fixed to underlying tissues. Reduced mouth opening with restriction of jaw movements was observed. Lips were competent. No cervical lymph nodes were clinically palpable, anteroposteriorly extending from the ala of the nose to 3 cm short of the tragus of the left ear (Figure 2).

**Figure 3:**
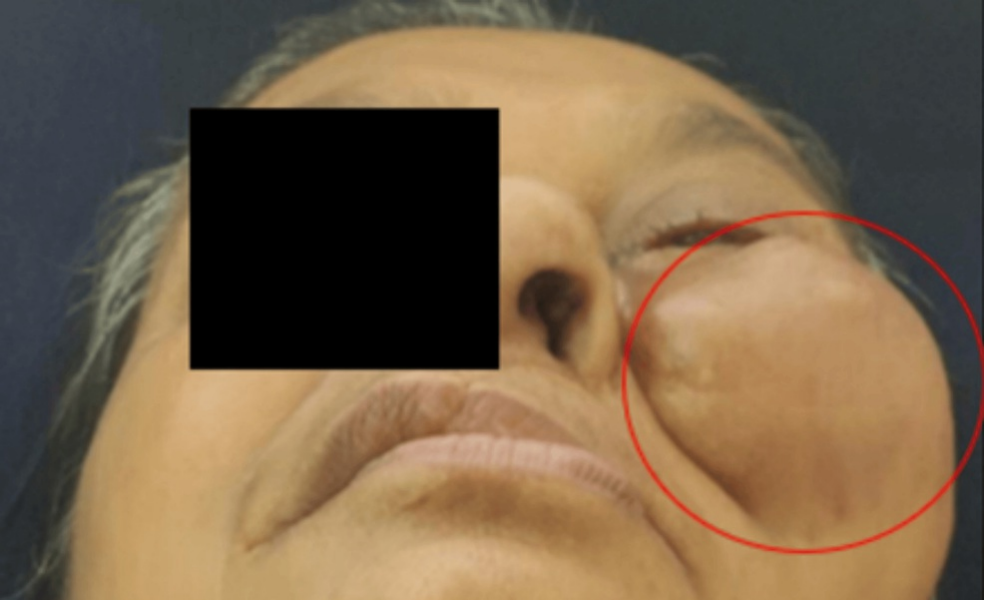
Worms view showing Left eye was distended superiorly due to swelling .

No abnormality was detected in the ear and nose. Intraoral examination revealed mouth opening of 2 mm approximately. Partial maxillectomy defect was noted. A single nonhealing ulceroproliferative lesion was present over the left posterior buccal mucosa extending anteroposteriorly, from the mesial of tooth number 36 (left first molar) to the retromolar trigone region on the left side. Supero-inferiorly from the depth of the upper buccal vestibule to the depth of the lower buccal vestibule on the left side. The size was 2 x 1 cm approximately, the shape was roughly oval, the edges were everted, the surface was irregular, the borders were ill-defined, and the color was whitish pink. The left eye was distended superiorly due to swelling (Figure 3).

**Figure 4:**
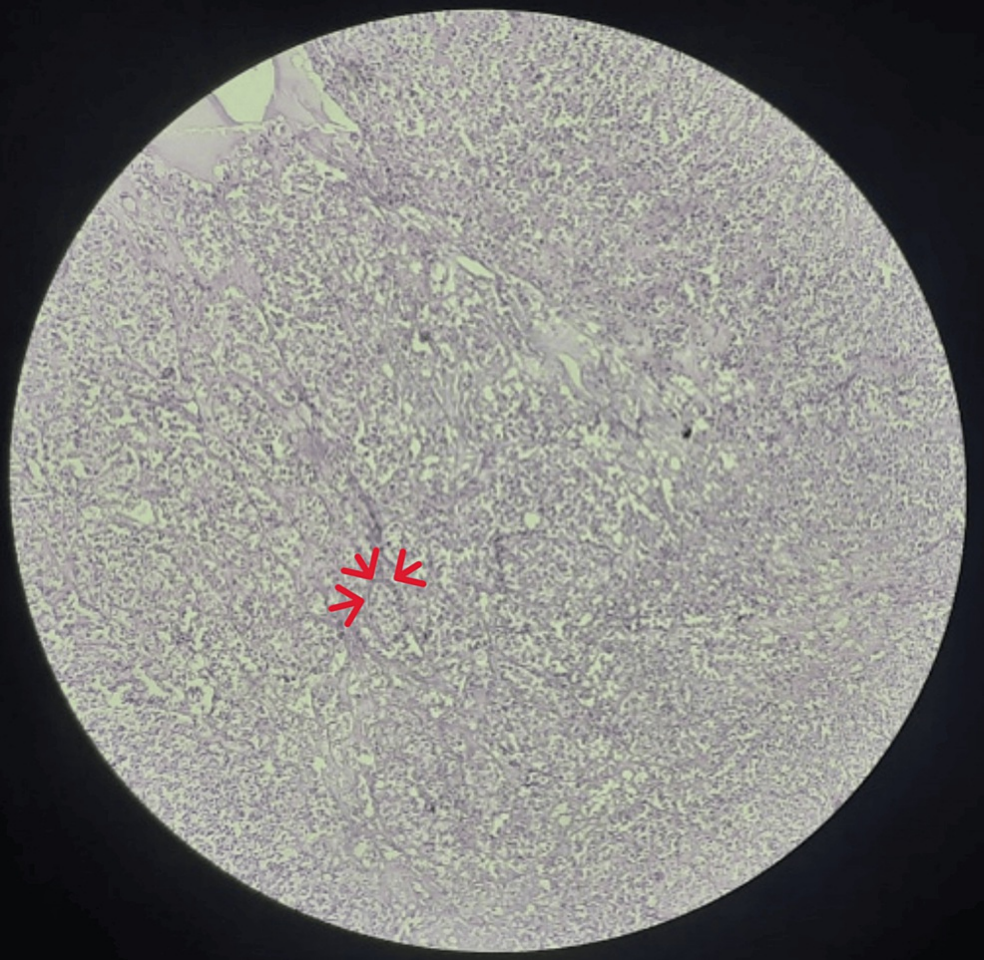
Histopathology showing both bilayer cells of the epithelial and myoepithelial components Biphasic arrangement of the inner luminal ductal cells and outer myoepithelial cells leads to epithelial-myoepithelial carcinoma diagnosis

**Figure 5:**
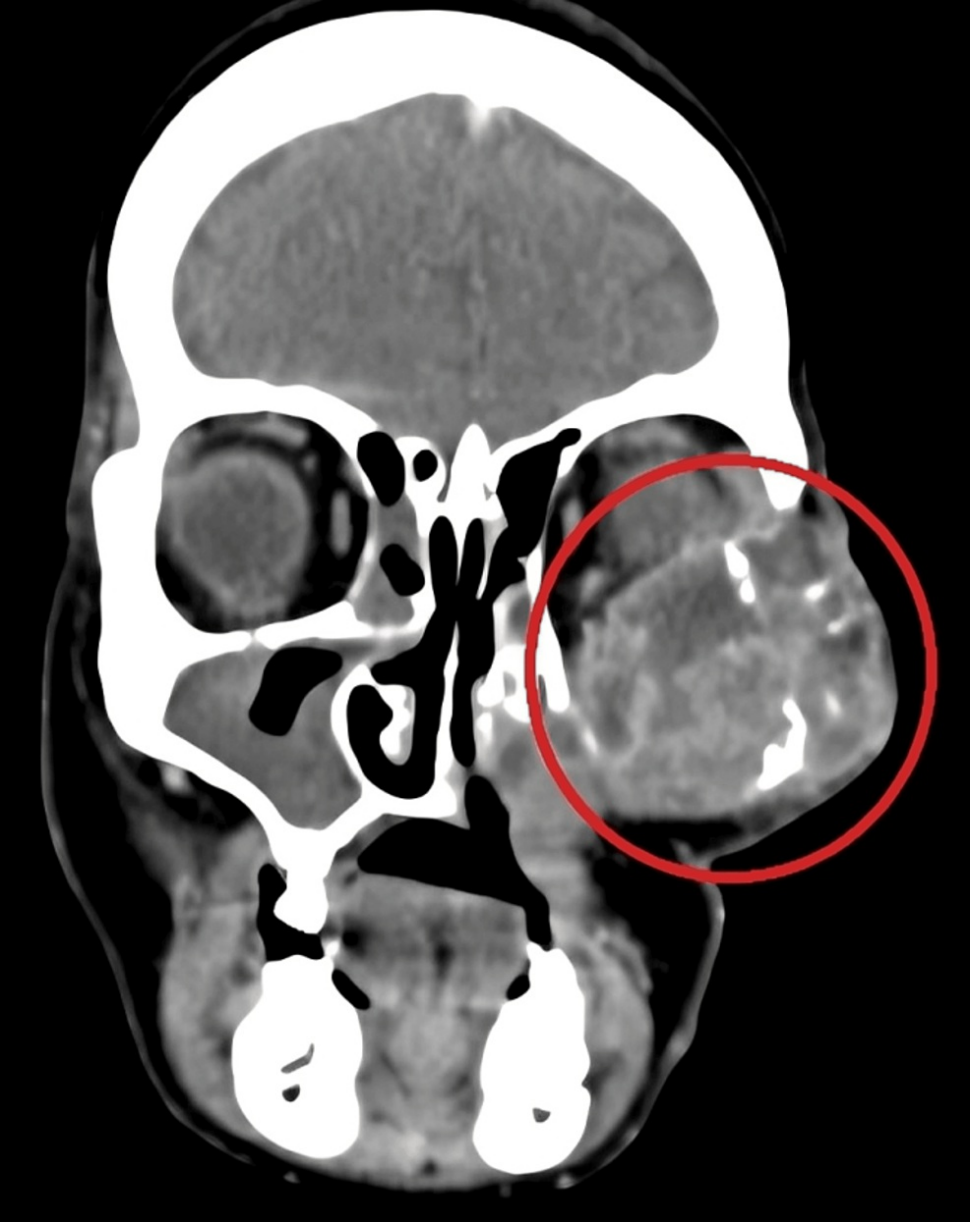
CECT coronal cut showing a heterogeneously enhancing necrotic mass lesion in the left maxillary region with infiltration into the adjacent temporalis muscle CECT: Contrast-enhanced computed tomography

**Figure 6:**
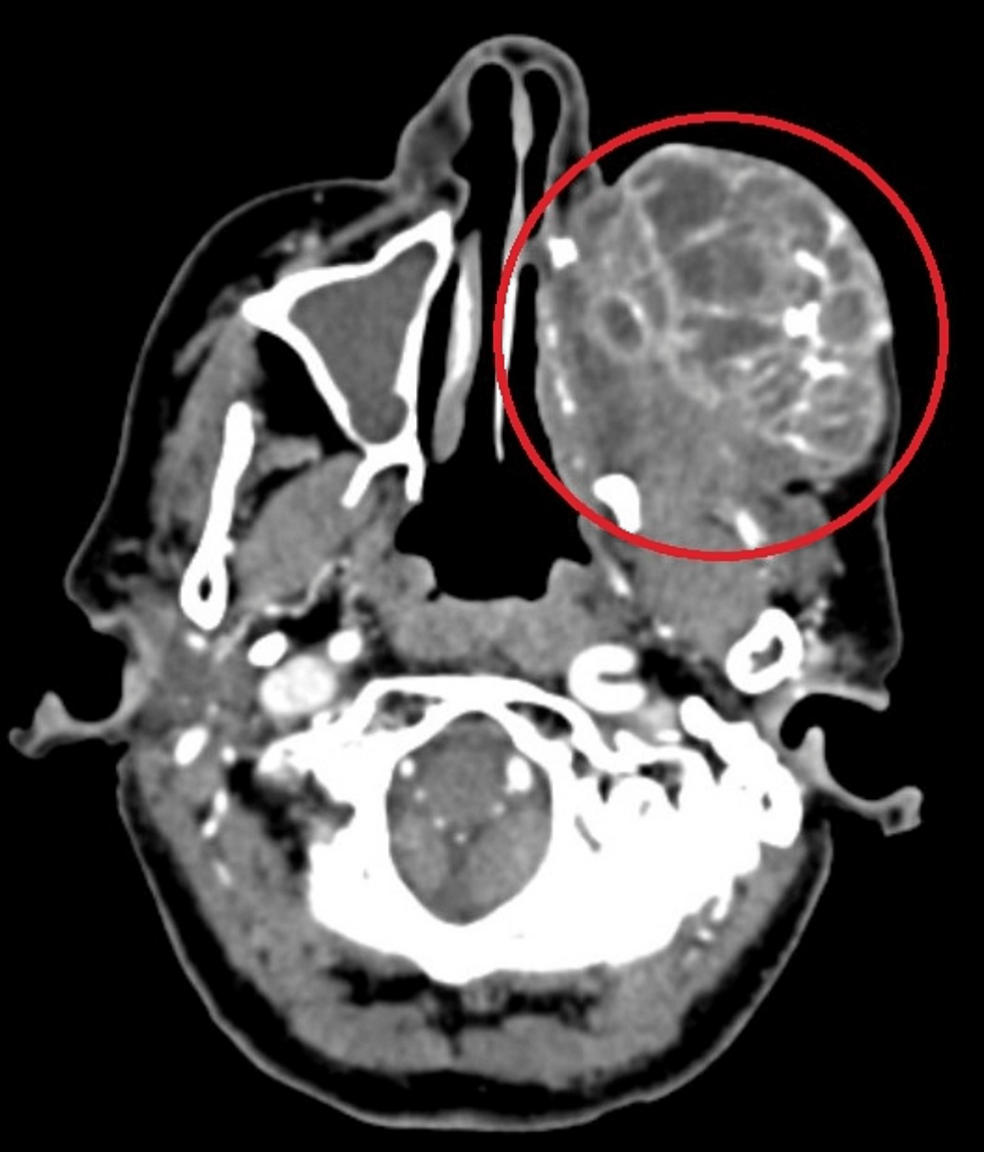
CECT axial cut showing mass abutting the inferior margin of the left globe CECT: Contrast-enhanced computed tomography

Histopathology showed features of EMC, consisting of both the epithelial and myoepithelial component (Figure 4). On contrast-enhanced computed tomography( CECT) investigation, reports were suggestive of heterogeneously enhancing necrotic mass lesion in the left maxillary region with infiltration into adjacent temporalis muscle and abutting the inferior margin of the left globe (Figure 5 and Figure 6).

**Figure 7:**
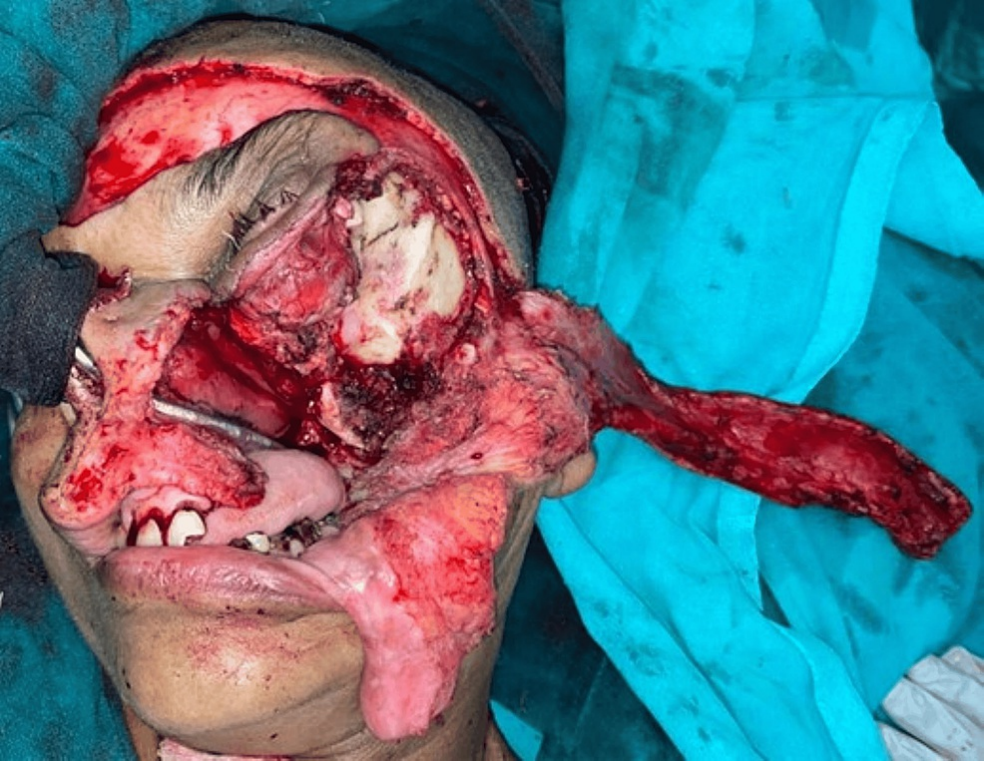
Surgical defect after maxillectomy till the pterygoid plates

Our treatment plan was composite resection of the lesion and reconstruction with forehead flap (Figure 7) showing the surgical defect after maxillectomy till the pterygoid plates.

**Figure 8:**
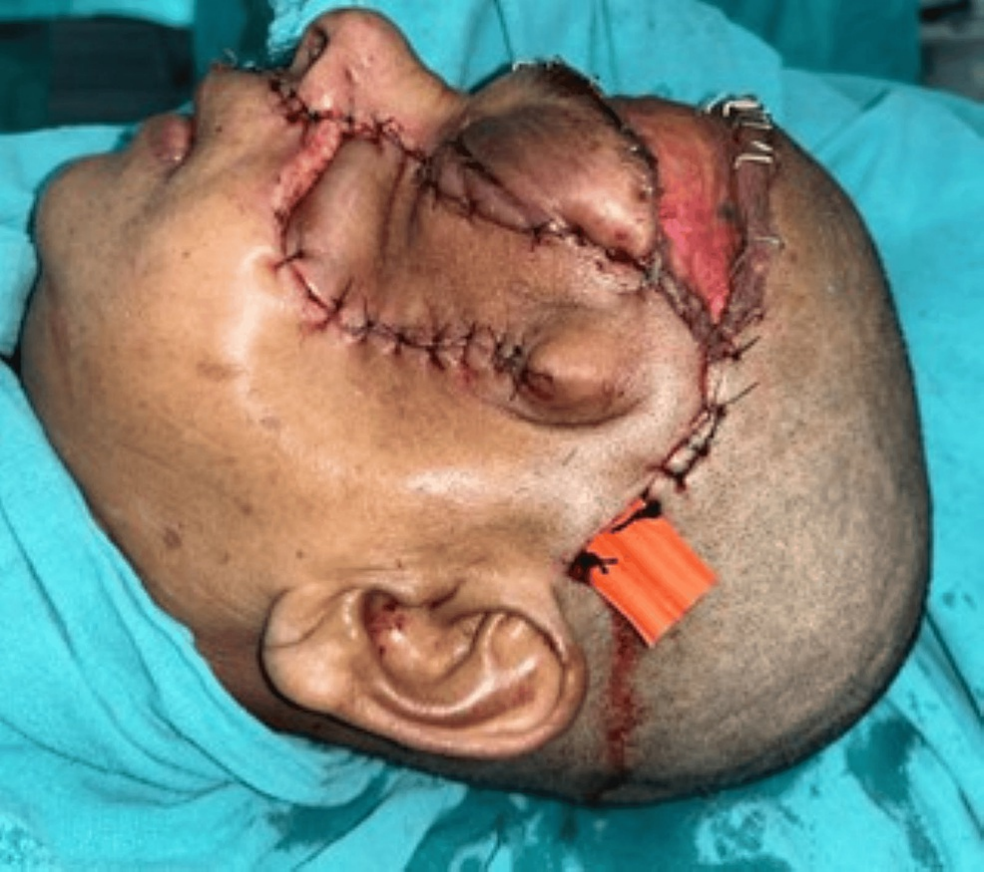
Closure of the surgical defect with a forehead flap

 Closure of the surgical defect was done with a forehead flap (Figure 8).

**Figure 9:**
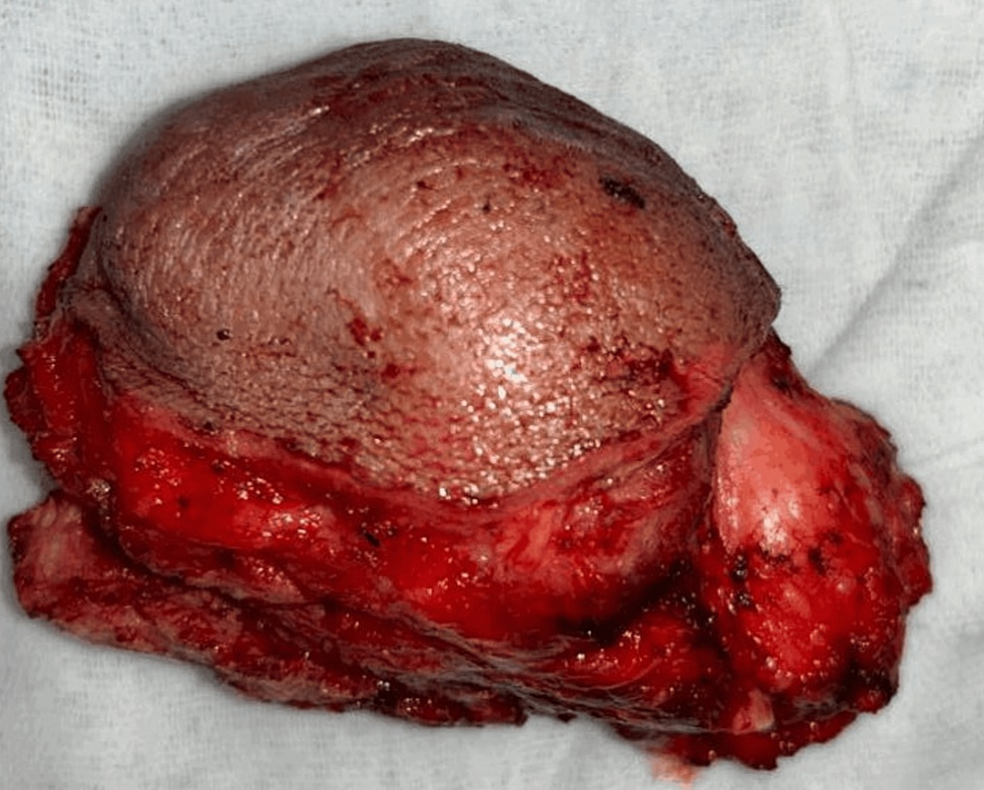
Resected specimen of the tumor

The resected specimen of the tumor was sent for further histopathological evaluation (Figure 9). The patient received radiotherapy postoperatively. The patient has not developed disease again in the one-year follow-up.

## Discussion

Studies reported a female gender-to-male gender ratio of 1.5:1, whereas 2:1 has been reported in majority of cases. Salivary gland (SG)-EMC was more common in females, and the reason behind this is unknown; genetics and hormonal factors such as androgens may be involved in the etiology of the lesion [6]. The differential diagnoses of the disease include adenoid cystic carcinoma, basal cell adenoma, basal cell adenocarcinoma, pleomorphic adenoma, myoepithelial carcinoma, and clear cell carcinoma [10].

It typically occurs in the sixth and seventh decades of life [11,12]. Due to the high degree of differentiation between cell types, the identification of tumors by histology is difficult. The tumor's morphology can resemble adenoid cystic carcinoma, clear cell carcinoma, or even pure myoepithelial carcinoma [13,14]. With a five- and 10-year illness-specific survival of 93.5% and 81.8% [6], 30%-50% of individuals experience local recurrence; lymph node and distant metastases are rarely seen [10]. EMC is recognized as a low-grade malignant tumor, with a common tendency for local recurrence following resection. This recurrence is frequently attributed to the incompleteness of the capsule, which typically confines this neoplastic formation. The occurrence of lymph node and hematogenous metastasis is less commonly observed [15]. It was discovered that mortality rates were elevated in cases where the tumor size exceeded 4 cm and when it exhibited a high-grade histology [16]. A subsequent examination of EMCs across various locations revealed that individuals with T2, T3, and T4 or M1 tumors experienced notably reduced survival compared to those with T1 or M0 tumors, respectively [17].

Thus, a thorough clinical examination which was suggestive of a single ulceroproliferative lesion present over the left posterior buccal mucosa extending from the mesial of the first molar on the left side to the retromolar trigone region on the left side. Supero-inferiorly it extended from the depth of the upper buccal vestibule to the depth of the lower buccal vestibule on the left side 2 x 1 cm approximately. Roughly oval, everted edges, irregular surface, ill-defined borders, whitish pink in color, a history of decreased salivation, and histopathological findings helped us arrive at the diagnosis.

The most effective course of action is thought to be surgical excision with a clear margin [18]. Thus, a clinical co-relation with clear recognition of histopathological features as given by definition of the WHO [19] guided in the arrival of diagnosis and proper treatment. Adjunctive radiation therapy is rarely advised, for example, in cases where surgical margins are difficult to define.

## Conclusions

In summary, a finding of a single ulceroproliferative lesion over the left posterior buccal mucosa, approximately 2 x 1 cm in size, roughly oval, everted edges, irregular surface, ill-defined borders, whitish pink in color, induration on palpation with a history of reduced salivation for 8-10 months approximately, change in saliva consistency from thin to thick ropy for 6-7 months approximately, and histopathological findings helped us arrive at the diagnosis. Our findings indicate that EMC is characterized as a low-grade carcinoma. The optimal diagnostic approach entails correlating clinical characteristics with histopathological observations while concurrently considering the potential differentials of EMC. Although metastasis is rare, local recurrence is notable. Thus, the recommended course of action for EMC involves meticulous surgical excision with adequate margins, followed by vigilant and prolonged postoperative surveillance.
